# The effects of health shocks on labor market outcomes: evidence from UK panel data

**DOI:** 10.1007/s10198-018-0985-z

**Published:** 2018-06-06

**Authors:** Otto Lenhart

**Affiliations:** Department of Economics, Duncan Wing, Strathclyde Business School, University of Strathclyde, 199 Cathedral Street, Glasgow, UK

**Keywords:** Health shocks, Labor market, Mechanisms, United Kingdom, C23, I10, I12, J60

## Abstract

This study examines the link between health shocks and labor market outcomes in the United Kingdom. For sample periods of up to 9 years, I use longitudinal data from the British Household Panel Survey to test how sudden health shocks affect a number of labor market outcomes, such as labor and household income, employment status, and hours worked. Additionally, the study examines potential mechanisms underlying the link between health declines and labor market outcomes. By estimating propensity score matching difference-in-differences models, the study shows that sudden health declines lead to significant and persistent reductions in earnings. The effects are strongest for individuals experiencing severe health shocks, males, individuals with higher education and those working in managerial jobs. When examining potential channels, I provide evidence that increased health care expenditures and health care usage as well as reduced work productivity can explain the observed effects on labor market outcomes.

**Electronic supplementary material:**

The online version of this article (10.1007/s10198-018-0985-z) contains supplementary material, which is available to authorized users.

## Introduction

Despite being previously examined by several researchers, there is still uncertainty about the relationship between labor market and health outcomes. While the majority of existing studies has focused on examining health effects following changes in income and employment, there has been growing interest in learning more about the effects of health on labor market outcomes in recent years. Given the close link between employment status and both health and health insurance coverage, an improved understanding of how labor market outcomes are affected following health shocks is relevant for policymakers. If health shocks have significant long-term negative spillover effects on employment and earnings, policymakers should discuss ways to provide improved assistance to workers experiencing sudden declines in health in dealing with the situation and allowing them to return to work. Additionally, there is little evidence about the potential mechanisms through which health shocks could affect immediate and long-term labor market outcomes. Using longitudinal data from the British Household Panel Survey (BHPS) for up to 9 years, this study examines the effects of health shocks on several labor market outcomes and explores potential channels through which labor market outcomes are affected following health shocks.

The analysis of this paper adds to the existing literature in two ways. First, the study uses longitudinal data from the UK. I am following the individuals over four different sample periods: 3, 5, 7 and 9 years. Besides testing for the immediate effects of health shocks on labor market outcomes, this also allows me to provide evidence whether people adapt to health shocks over time or whether the effects are persistent. These findings could indicate whether there are potential labor market institutions in place that make it challenging to reintegrate workers into the workforce following the health issues.

Second, besides testing for the presence of a causal link from health to labor market outcomes, this study examines potential mechanisms through which health shocks can affect labor market outcomes. An examination of potential channels is important from a policy and a social welfare perspective since it provides evidence for how policymakers can potentially help prevent substantial losses in productivity following health shocks. This study examines the role of three mechanisms: (1) changes in the frequency of health care usage. This can impact labor market outcomes by taking time away from work and work-related activities; (2) changes in the likelihood of paying for health care. While universal health coverage is provided in the UK by the National Health Service (NHS), individuals have the option to purchase additional private care to forego long waiting times and receive potentially lower quality care; (3) changes in the worker’s productivity. Observing changes is the productivity of workers who previously experienced negative health shocks could suggest that employees should find ways on how to allow workers to be better reintegrated to the workforce, while policymakers should create an environment that mitigates the risks to employees experiencing sudden health shocks.

The study finds that health shocks, captured by declines in self-reported health status and the onset of health conditions, significantly affect labor market outcomes. Negative health events are shown to reduce labor earnings, household income, and the likelihood of being employed. The negative effects are found for all four variations of the sample length, which suggests that health shocks have lasting impacts on labor market outcomes. When examining the effects across different subgroups of the population, I find larger effects for males, higher educated individuals, and those working managerial and professional jobs. When examining potential mechanisms, the study provides evidence that increased health care usage and health care expenditures as well as reduced work productivity can explain the observed persistent negative effects of health shocks on labor market outcomes.

## Labor market outcomes and health: previous evidence

A number of previous studies have examined health effects of negative employment shocks on people’s health. It has been established that negative employment events such as mass layoffs, plant closings and job loss have significant negative effects on health outcomes of affected individuals [20, 21, 37, 52, 53, 56]. Furthermore, other studies have examined the association between worsened economic conditions and health outcomes. These findings are mixed: earlier work provides evidence that economic downturns actually improve health outcomes [40, 44, 51], while more recent work suggest that health declines along with the economy [15, 38, 39].

A large number of studies have examined the relationship between income and health. Following the pioneering study by Case et al. [10], several papers have also provided evidence for the presence of a strong positive association between household income and health [4, 13, 14, 34, 47, 48]. This phenomenon has become known as the income gradient in health. In more recent year, researchers have expressed the need to test for the causal nature between income and health by pointing out that the presence of a positive association could be the result of third factors that influence both health and income or due to reverse causality, which exists if health outcomes influence people’s employment status and, therefore, their income.

Several previous studies have examined the effects of health shocks on labor market outcomes. The majority of early work on the topic has focused on elderly groups of the population and the effects of health on retirement [7, 9, 18, 26, 31, 32, 49, 54, 60]. These studies established that older adults are significantly less likely to be employed and more likely to retire following the occurrence of a health shock. Other studies additionally show that health shocks have negative labor market effects for younger individuals by examining several types of health shocks. These include the presence of permanent health conditions [46], reduced psychological health [12], injuries from road accidents [16], the onset of disability [11, 36], reduced physical health [22, 23] and sudden illness [24].

Van Doorslaer and Koolman [58] find that income-related health inequalities in the UK are larger than in most other European countries, while García-Gómez et al. [24] suggest that differences in the provision of disability benefits could explain these differences across nations. The authors argue that relating the size of benefits to previous earnings, as done in the Netherlands, reduces the average income loss from health shocks compared to when benefits are paid at a flat rate like in the UK. Besides examining the effects of negative health events on labor market outcomes, this study tests for additional potential mechanisms that can explain health-related inequalities in the UK (health care usage, health care expenditures, and work productivity).

## Data

This study uses data from waves 10–18 (2000–2008) of the British Household Panel Survey (BHPS), a nationally representative panel survey of private households in Great Britain that started interviewing 10,300 individuals from 5,500 families in 1991.^1^ The use of the BHPS provides several advantages for the purpose of this study. Due to it longitudinal nature, the dataset allows accounting for time-invariant unobserved heterogeneity and compositional selection. The potential for measurement error in the self-reported health measure is reduced since each individual’s health is only compared to his or her own prior assessment. This allows controlling for the fact that each respondent may have their own scales in ranking their health (reference bias). Furthermore, in comparison to the two other commonly used UK datasets with detailed information on labor market outcomes (Labor Force Survey and New Earnings Survey), the BHPS also provides information on several health outcomes. Finally, the BHPS gives a complete representation of incomes across the pay distribution since it questions all individuals above 15 years of age who live in the household at the time of the interview. Given that individuals in the UK become eligible to receive state pensions at the age of 65, the sample is restricted to all individuals between the ages 18 and 64 in the surveyed households for whom information on health and labor market outcomes is available.

The analysis uses two different types of health shocks. In the main specification, which is presented in Table 1, health shocks are defined as a decline in self-reported health status. Self-assessed health is categorized from 1 (= excellent) to 5 (= very poor) in the BHPS. This measure of health has been shown to be a good predictor of other health outcomes, including mortality [29], future health care usage [57] and hospitalizations [45]. To remove concerns about potential reporting heterogeneity of health status, Johnston et al. [33] suggest the additional use of more objective health outcomes. In an additional specification, health shocks are defined as the onset of a new health condition. In each wave, the BHPS asks respondents whether they suffer from any of the following 15 health conditions: body pain, migraine, skin issues/allergy, asthma/chest pain, anxiety, heart or blood pressure, hearing problems, stomach/liver/kidney pain, seeing problems, epilepsy, diabetes, alcohol or drug problems, stroke, cancer or other conditions. As indicated by García-Gómez [23], using the onset of health conditions as a health shock could provide evidence regarding any potential anticipation effects [23].

**Table 1:** DD model setup

	Treatment group		Control group	
	Health	Employment	Health	Employment
*Panel A*				
3-year				
2000 (pre)	Excellent/very good	Working	Excellent/very good	Working
2001 (shock)	Fair/poor/very poor	Working	Excellent/very good	Working
2002 (post)	Fair/poor/very poor		Excellent/very good	
*Panel B*				
5-year				
2000 (pre)	Excellent/very good	Working	Excellent/very good	Working
2001 (pre)	Excellent/very good	Working	Excellent/very good	Working
2002 (shock)	Fair/poor/very poor	Working	Excellent/very good	Working
2003 (post)	Fair/poor/very poor		Excellent/very good	
2004 (post)			Excellent/very good	
*Panel C*				
7-year				
2000 (pre)	Excellent/very good	Working	Excellent/very good	Working
2001 (pre)	Excellent/very good	Working	Excellent/very good	Working
2002 (pre)	Excellent/very good	Working	Excellent/very good	Working
2003 (shock)	Fair/poor/very poor	Working	Excellent/very good	Working
2004 (post)	Fair/poor/very poor		Excellent/very good	
2005 (post)			Excellent/very good	
2006 (post)			Excellent/very good	
*Panel D*				
9-year				
2000 (pre)	Excellent/very good	Working	Excellent/very good	Working
2001 (pre)	Excellent/very good	Working	Excellent/very good	Working
2002 (pre)	Excellent/very good	Working	Excellent/very good	Working
2003 (pre)	Excellent/very good	Working	Excellent/very good	Working
2004 (shock)	Fair/poor/very poor	Working	Excellent/very good	Working
2005 (post)	Fair/poor/very poor		Excellent/very good	
2006 (post)			Excellent/very good	
2007 (post)			Excellent/very good	
2008 (post)			Excellent/very good	

Besides examining the short- and long-run effects of health shocks on earnings and employment, this study additionally tests for the role of potential mechanisms underlying the link between sudden declines in health and labor market outcomes. According to the Grossman [25], health can be viewed as both a consumption and investment good since it not only makes people feel better, but it also increases the number of healthy days to work and to earn income. Grossman [25] states that to keep certain levels of health capital, individuals invest into their health through channels such as market inputs of health care, diet and exercise.

The first mechanism examined is changes in health care usage, which is captured by three outcomes: (1) the likelihood of having more than five annual doctor visits, (2) the likelihood of having spent a night at a hospital in the previous year, (3) the likelihood of having used a number of other health services, and (4) changes in the likelihood of having paid for health care services in the previous year. While the NHS provides universal coverage to all individuals in the UK, two serious issues that the program has been dealing with are the quality of care and long waiting times [59]. To avoid these problems, individuals have the option to purchase additional private care to forego long waiting times before seeing a doctor in some cases. Persistent increases in out-of-pocket expenditures on health can be associated with labor market outcomes in two ways: (1) earned income of individuals recovering from health shocks could be reduced due to time spent away from work for doctor visits, and (2) household income could be affected to a larger extent than individual income if other household members take time off from work to help their family member with the doctor appointments. Given that the analysis uses four different sample lengths, it is able to provide evidence whether any potential changes in health care usage only occur immediately after the health shock or whether these changes persist for several years.

While health shocks can reduce the labor force participation of people who can no longer work, another channel through which adverse health events can affect earnings is by reducing labor productivity. Workers might not be able to perform the same tasks or might need longer to complete the same tasks compared to before the health shock. Without empirically testing for the presence of this channel, García-Gómez and López-Nicolás [22] point out that productivity losses could either be absorbed by the employer or by the inability to work extra time. Using a sample of 2264 workers, Myde et al. [43] provide evidence for a strong link between health risks and self-reported work productivity.

To capture whether changes in work productivity are a potential mechanisms underlying the relationship between health shocks and labor market outcomes, this analysis examines four proxies for work productivity: (1) hourly wages of workers, (2) the likelihood of reporting that current work is limited by one’s health, (3) the likelihood of having difficulty to concentrate, and (4) the likelihood of constantly feeling under strain. While hourly wages is the most direct way of measuring productivity, the other three outcomes should provide additional evidence for potential changes in labor productivity following the onset of health shocks. Antikainen and Lönnqvist [5] suggest that, especially in “knowledge-intensive” organizations, where knowledge has more importance than other inputs, work performance can be negatively affected by health problems or other personal issues because they are highly dependent on the ability to concentrate. Using a factor analysis model, Halkos and Bousinakis [27] provide empirical evidence that increased stress leads to reduced work productivity. Using the four proxies of work productivity listed above, my study tests whether individuals who suffered health shocks are not able to perform the same tasks compared to prior to the experiencing the sudden health decline.

## Econometric methods

### DD matching models

Similar to García-Gómez and López-Nicolás [22], this study estimates propensity score matching combined with difference-in-differences (DD) models to estimate the Average Treatment Effect on the Treated (ATET). This empirical strategy allows me to compare the distributions of outcomes for treated individuals (who suffer the health shock) with the distributions of outcomes of matched individuals in the control group, without having to make any functional form assumptions. As pointed out by García-Gómez and López-Nicolás [22], matching frameworks are often criticized for assuming away potential biases that might exist due to unobserved heterogeneity. The authors argue that one solution to remove concerns about such biases is the use of longitudinal data that includes data from before and after the health shock. Using longitudinal data from the BHPS, this study is able to first difference the outcomes of the treated and the controls to eliminate any unobservable fixed effects that influence the selection into the groups as well as the outcomes of interest [22]. Thus, the estimated ATET’s are weighted averages of the differences in differences between each of the treated individuals and his/her matched control.

The study uses estimated propensity scores, which calculate the probability of treatment given a vector of observable variables, to match individuals who receive a health shock to individuals that are similar but do not experience the health shock. The propensity scores are based on pre-treatment variables and are estimated using probit models. Observable characteristics that are included to obtain the propensity scores are age, gender, household size, educational attainment, and area. In additional specifications, I furthermore include information on lagged health status as a covariate when estimating the propensity scores. Following Rosenbaum and Rubin [50], the use of a function of X, called the propensity scores P(X), rather than a potentially high-dimensional vector of covariates implies that:

1 $$E{\text{ }}\left( {{Y_0}|{\text{ }}D{\text{ }}={\text{ }}1,{\text{ }}P\left( X \right)} \right){\text{ }}={\text{ }}E{\text{ }}\left( {{Y_0}|{\text{ }}D={\text{ }}0,{\text{ }}P\left( X \right)} \right),$$

where *Y*_0_ denotes the untreated state, *D* = 1 indicates treatment and *D* = 0 indicates non-treatment. The analysis of this study follows Heckman et al. [28] difference-in-difference matching methods, which uses both comparisons between treated and non-treated, and differencing over time. Thus, the conditions needed to identify the ATET using the difference-in-difference matching estimator is:

2 $$E{\text{ }}\left( {{Y_{0,t}}-{\text{ }}{Y_{0,{t^\prime }}}|{\text{ }}D{\text{ }}={\text{ }}1,{\text{ }}P\left( X \right)} \right){\text{ }}={\text{ }}E{\text{ }}\left( {{Y_{0,t}}-{\text{ }}{Y_{0,{t^\prime }}}|{\text{ }}D{\text{ }}={\text{ }}0,{\text{ }}P\left( X \right)} \right),$$

where *t* and *t*′ represent the post- and pre-treatment periods, respectively. Thus, the ATET provides a weighted average of the differences in differences between individuals in the treatment and the control group and it is obtained by estimating the following equation:

3 $${\text{ATE}}{{\text{T}}_{{\text{DID}}}}={\text{ }}E\left( {{Y_{1t}}|D=1,{\text{ }}P\left( X \right)} \right){\text{ }}-{\text{ }}E\left( {{Y_{0t}}|D=0,{\text{ }}P\left( X \right)} \right){\text{ }} - {\text{ }}E\left( {{Y_{1{t^\prime }}}|D=1,{\text{ }}P\left( X \right)} \right){\text{ }}-{\text{ }}E\left( {{Y_{0{t^\prime }}}|D=0,{\text{ }}P\left( X \right)} \right)$$

In the empirical analysis, the study uses two alternative methods when matching treated individuals with those in the control group [8]: (1) nearest neighbor matching on the propensity score, and (2) kernel matching on the propensity score. Since there are no reasons to expect one of the methods to be preferable to the other, the use of both methods allows the analysis to test for the robustness of the observed effects. When examining the ATET of health shocks on labor market outcomes, the analysis examines four different outcomes: (1) total annual labor income, (2) total annual household income, (3) the probability of being employed, and (4) hours worked per week. Standard errors are obtained following recent findings by Abadie and Imbens [3], who established how to take into account that propensity scores are estimated in the first stage. They show that ignoring this fact when estimating average treatment effects on the treated in the second stage may lead to confidence intervals that are either too large or small.^2^ By showing that the propensity matching estimator have approximately Normal distributions, Abadie and Imbens [3] provide evidence that the matching on estimated propensity score is more efficient than matching on the true propensity score in large samples.

### Assignment of treatment and control groups

This assignment of individuals into treatment and control group used in this study is similar to previous work by García-Gómez and López-Nicolás [22] and García-Gómez [23] as well as Lechner and Vázquez Álvarez [35]. A crucial challenge when estimating the effects of health shocks on labor market outcomes is the fact that many health and labor market outcomes are potentially jointly determined by many people. The use of propensity score matching DD model can overcome this concern by identifying arguably exogenous health shocks that are independent of employment status. Table 1 shows the setup for the two groups used in the DD models estimated in this study, which analyzes four variations of sample length to test for both immediate and the long-term effects of adverse health events on labor market outcomes. Despite different sample periods, all three models share the following characteristics:

1. Individuals from both treatment and control group are in excellent or very good health and are working in the pre-treatment period (Pre).
2. Individuals forming the treatment group experience a health shock in the treatment period (Shock), meaning their health status drops to fair, poor or very poor. Individuals in the control group remain in excellent or very good health. All members of the treatment and the control group are working during the period in which the treatment occurs.
3. Self-reported health status of individuals in the treatment group remains in fair, poor or very poor health in the first year after the health shock, while individuals in the control group remain in excellent or very good health throughout the post-treatment period (Post).

Additionally, using the same setup as shown in Table 1, I use the onset of a health condition as an alternative health shock. Individuals forming the treatment group report the onset of a health condition in the treatment period, while those in the control group report no health conditions throughout the study period. Again, all individuals work in both the pre-treatment and the treatment period. Given that information regarding the presence of health conditions are potentially less subjective than self-assessed health status, the findings from this additional health shock can remove concerns about potential reporting heterogeneity of health status [33].

By examining a sample of individuals who are employed during both the pre-treatment period and the year of the health shock, the potentially simultaneous determination of health and labor market outcomes is accounted for and allows testing for the effects of experiencing health declines on labor market outcomes in the post-treatment period. This framework ensures that the observed effects are not the result of reverse causality, which would exist if changes in labor market outcomes lead to the health shock. One assumption of this framework is that there are no anticipation effects, meaning that people report declines in health because they expect negative employment shocks to occur in the future.

### Descriptive statistics

Table 2 presents sample sizes for the four different sample length for each of the two health shocks that examined in the study. For the health status shock in the shortest period of study (2000–2002), the control group consists of 9720 observations and the analysis includes 591 observations for the treatment group. The analysis includes only individuals that are present in all waves of the corresponding sample periods. Given that the onset of new health conditions occur less frequently than declines in health status, the sample sizes are smaller when using health condition as the health shock.

**Table 2:** Sample sizes for treatment and control group

	Health shock: drop in health status			Health shock: onset of health condition		
	Treated	Control	Total	Treated	Control	Total
3-year sample	591	9720	10,311	1620	5034	6654
5-year sample	585	11,155	11,740	1525	5190	6715
7-year sample	504	11,760	12,264	1001	5061	6062
9-year sample	315	11,268	11,583	432	4635	5067

Given that the analysis in this study only includes individuals who are present in all survey waves for each sample length period (either 3, 5, 7 or 9 years), attrition could pose a potential issue. The obtained treatment effect estimates could potentially be biased if people drop out of the survey due to health-related problems. Given the longitudinal data set of the BHPS, I am able to identify individuals who drop out of the survey and compare them to those who remain in it and are used in the analysis. Online Appendix Table A1 shows comparisons of descriptive statistics for the two groups for the sample periods of 5, 7, and 9 years. It is noticeable that there are only very small differences in health status between people remaining in the survey and those who drop out at some point. Individuals who stay in the BHPS for all the years in each of the three period analyzed are shown to be slightly more likely to be employed and have higher labor and household incomes than those who drop out of the survey. Table A1 shows that the attrition rates were between 13 and 15% for the sample periods shown in Online Table A1.

**Fig. 1:**
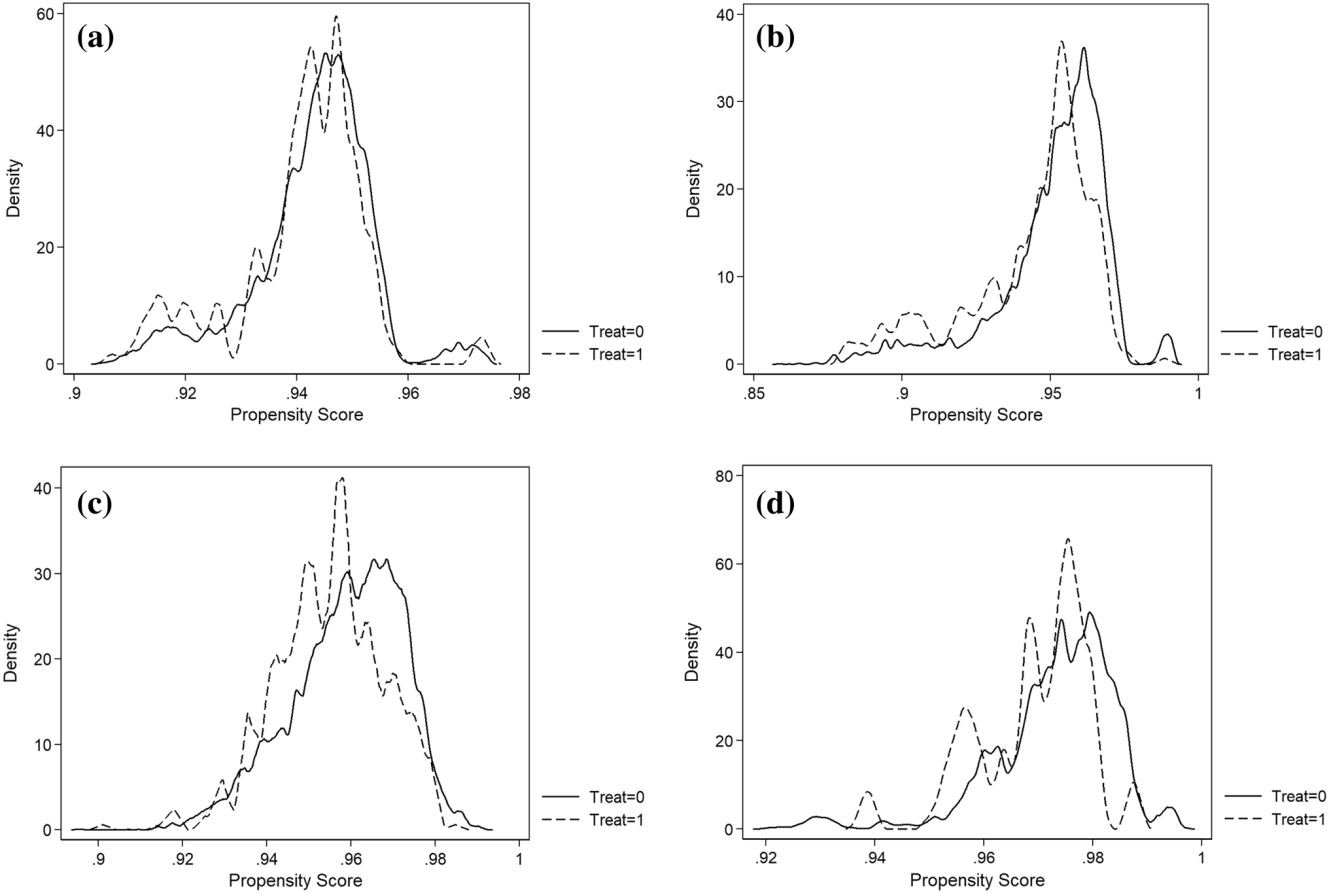
Density of propensity scores, **a** 3-year sample, **b** 5-year sample, **c** 7-year sample, **d** 9-year sample

One of the main assumptions of estimating propensity score matching DD estimates is that the overlap assumption, which is satisfied when there is a chance of seeing observations in both control and treatment groups at each combination of covariate values. As highlighted in the reference manual for Stata Treatment Effects, the analysis cannot predict or otherwise account for the unobserved outcomes of some individuals if the assumption is violated [55]. Figure 1a–d provide plots of the estimated densities of the propensity scores for both treatment and control groups for all four sample periods. All the graphs present clear evidence that the overlap assumption is satisfied since there are chances of seeing observations in both groups at each combination of covariate values.

Table 3 provides results from covariate balance tests for the matching conducted in the analysis. In well specified matching models, the covariates should be balanced, which allows for the outcomes to be conditionally independent of the treatment when conditioning on covariates [55]. The left side of Table 3 shows the balance test results for the main analysis, while the right side shows differences between raw and matched data when lagged health information is included as a covariate.^3^ Overall, the balancing results indicate that the matching succeeds in balancing the covariates and reducing the standardized differences between the two groups.

**Table 3:** Covariate balance tests

	Main analysis				Analysis with controls for lagged health			
	Standardized differences		Variance ratio		Standardized differences		Variance ratio	
	Raw	Matched	Raw	Matched	Raw	Matched	Raw	Matched
3-year								
HH size	− 0.0175	0.0024	0.9420	0.9163				
Education	0.1734	− 0.0018	0.9906	0.9687				
Age	0.1071	− 0.0186	1.0413	1.0315				
Gender	− 0.0022	0.0275	1.0014	1.0016				
Area	− 0.0335	− 0.0127	0.9285	0.9490				
5-year								
HH size	− 0.0672	0.0417	1.0414	1.1571	− 0.0672	− 0.0310	1.0414	1.1079
Education	0.3427	0.0149	0.9780	0.9559	0.3427	− 0.0757	0.9780	0.9161
Age	0.1533	0.0432	0.9215	0.8796	0.1533	0.0243	0.9215	0.8692
Gender	0.1038	− 0.0715	0.9996	0.9896	0.1038	− 0.0017	0.9996	0.9999
Area	− 0.0769	− 0.0320	1.0018	0.9967	− 0.0769	− 0.1255	1.0018	0.9490
Lagged health status	–	–	–	–	0.6766	0.0187	0.6010	0.9708
7-year								
HH size	− 0.1542	0.0182	1.1199	1.2669	− 0.1542	− 0.2428	1.1199	0.9570
Education	0.1278	− 0.0738	0.9103	0.8231	0.1278	0.4209	0.9103	1.3985
Age	− 0.0831	− 0.0123	1.0280	1.0092	− 0.0831	0.1321	1.0280	1.1516
Gender	− 0.0428	− 0.0892	1.3490	1.9217	− 0.0428	− 0.2305	1.3490	1.1363
Area	0.2269	0.0432	0.7737	0.8212	0.2269	− 0.0232	0.7737	0.7864
Lagged health status	–	–	–	–	0.7242	− 0.0782	0.5197	1.0818
9-year								
HH size	0.0455	0.0744	0.8414	0.7221	0.0455	0.0440	0.8414	0.9859
Education	0.2734	− 0.0498	1.0289	1.1293	0.2734	0.0232	1.0289	0.8383
Age	0.1377	0.0050	0.8731	0.8611	0.1377	− 0.1599	0.8731	1.0662
Gender	− 0.0170	− 0.0145	1.4092	2.0770	− 0.0170	0.0592	1.4092	0.9511
Area	0.2399	0.0667	0.8979	0.9703	0.2399	0.2130	0.8979	0.8791
Lagged health status	–	–	–	–	0.7109	− 0.0410	0.6144	0.8386

## Results

### The effects of health status shocks

Table 4 presents propensity score matching DD effects of sudden declines in health status on four labor market outcomes and four different sample lengths. The estimates in the first two columns provide evidence that health shocks have substantial negative effects on labor earnings of affected individuals. The nearest neighbor matching estimates find reductions in annual labor income in the range of £1181.40 for the year after the shock in the 3-year sample (*p* < 0.01) to £4432.32 for the 9-year sample (*p* < 0.01), which examines the effects for up to 4 years after the health shock.^4^ The immediate effects on earnings in the year following the drop in health status is slightly smaller in magnitude than estimated by García-Gómez and López-Nicolás [22], who find a decline in income of €1118 (measured in 2001 Euros, which corresponds to £1763.31 using the 2001 €/£ conversion rate) using Spanish data and a 3-year sample.

**Table 4:** Effects of health shocks on labor market outcomes (health status)

	Total labor income (£ per year)		Total HH income (£ per year)		Employed		Weekly work hours	
	NN matching	Kernel matching	NN matching	Kernel matching	NN matching	Kernel matching	NN matching	Kernel matching
3 year sample	− 1181.40*** (430.54)	− 769.08 (621.24)	− 2834.63*** (756.41)	− 3355.70*** (1065.85)	− 0.0068 (0.0073)	− 0.0186* (0.0109)	− 0.06 (0.57)	− 1.14 (0.72)
5-year sample	− 3041.75*** (462.60)	− 3948.23*** (752.80)	− 4362.41*** (777.40)	− 4255.36*** (1063.76)	− 0.0356*** (0.0103)	− 0.0370*** (0.0149)	− 1.17** (0.58)	− 0.57 (0.65)
7-year sample	− 2097.46*** (437.16)	− 671.85 (720.86)	− 3025.02*** (715.66)	− 4677.16*** (1345.02)	− 0.0378*** (0.0128)	− 0.0268* (0.0149)	0.93 (0.57)	0.39 (0.78)
9-year sample	− 4432.32*** (810.96)	− 3345.17** (1583.87)	− 5005.84*** (842.24)	− 4871.36*** (1683.60)	− 0.0052 (0.0071)	− 0.0159*** (0.0052)	0.35 (1.07)	0.51 (0.88)

Robust standard errors, based on Abadie and Imbens [1], are shown in parentheses. Income is adjusted for inflation, using the UK. Consumer price Index and 2000 as the base year

*p < 0.10, **p < 0.05, ***p < 0.01

While the kernel matching estimates also provide evidence for declines in labor income in all four periods, two of the effects are statistically insignificant. Overall, given that losses in labor income are larger in magnitude for the longer sample periods, the results do not suggest that individuals adapt to the health shock. On the contrary, it appears that individuals struggle to be reintegrated into the labor force following the declines in health. The next two columns show the effects on health declines on annual household income. For all sample periods and both matching techniques, I find even larger reductions in household income than for labor income. For the 9-year sample, the results suggest that household incomes are reduced by £5005.84 and £4871.36 following the health shock (both *p* < 0.01). A potential explanation for the difference in magnitudes for total household income and individual labor earnings is that other household members take time away from work to assist the individuals recovering from health shocks.

Appendix Table A2 furthermore provides matching DD results for the effects of sudden declines in health status on the natural log of both total labor and household income. Consistent with the results in Table 4, all estimates show that health shocks negatively affect labor earnings and total household incomes of affected individuals. While it should be noted that two of the eight labor income estimates are imprecisely estimated, Online Table A2 confirms that the observed treatment effects are robust to the measure of income used in the analysis.

Table 4 additionally shows the effects of health shocks on the likelihood of being employed and weekly hours worked. My analysis finds that individuals reduce their labor market activity on the extensive margin, while there is little evidence that there are any changes on the intensive margin of employment. While four of the eight estimates for the likelihood of being employed are statistically significant at the 1% level, the immediate effects are substantially smaller than those observed by García-Gómez and López-Nicolás [22] for Spain. When re-estimating the analysis with only individuals who remained at work throughout the sample periods, I find very similar declines in labor earning and household income. This suggests that changes in employment are not the only driver of the observed income losses. The later part of the study examines some other potential mechanisms through which health shocks can affect labor market outcomes.^5^

### Annual treatment effects

Table 5 shows annual estimates for the effects of health shocks on total annual labor income and the likelihood of being employed, which are obtained by interacting each year with the treatment indicator. Since this test includes effects during pre-shock periods, it provides a test for the parallel trends assumption made in the main DD model. Given that this analysis is not feasible in the 3-year sample, Table 5 only shows treatment effects for sample periods of 5, 7 and 9 years.

**Table 5:** Annual treatment effects

	Total labor income (£ per year)	Employed
5-year sample (health shock in 2002)		
Treat*2000	456.45 (571.33)	0.0089 (0.0182)
Treat*2001	29.13 (548.56)	0.0056 (0.0152)
Treat*2003	− 496.32 (526.55)	0.0017 (0.0192)
Treat*2004	− 1913.57** (763.25)	− 0.0734*** (0.0310)
7-year sample (health shock in 2003)		
Treat*2000	695.75 (873.84)	− 0.0090 (0.0236)
Treat*2001	124.97 (762.76)	− 0.0083 (0.0194)
Treat*2002	1336.91 (846.87)	0.0063 (0.0213)
Treat*2004	− 1358.27* (781.94)	0.0568 (0.0367)
Treat*2005	− 2078.62** (923.18)	− 0.0572 (0.0361)
Treat*2006	− 2498.40** (1022.74)	− 0.1212*** (0.0431)
9-year sample (health shock in 2004)		
Treat*2000	592.78 (2527.47)	− 0.0005 (0.0077)
Treat*2001	882.40 (2567.48)	0.0004 (0.0048)
Treat*2002	2161.92 (2325.20)	0.0021 (0.0054)
Treat*2003	128.95 (2301.63)	− 0.0009 (0.0057)
Treat*2005	385.63 (2226.96)	0.0145 (0.0259)
Treat*2006	− 948.35 (2033.88)	− 0.0198*** (0.0063)
Treat*2007	− 1551.61 (2298.16)	− 0.0319*** (0.0066)
Treat*2008	− 1625.28 (2229.07)	− 0.0421*** (0.0071)

Robust standard errors, clustered by individuals and based on Abadie and Imbens [1], are shown in parentheses. Income is adjusted for inflation, using the UK. Consumer price Index and 2000 as the base year

*p < 0.10, **p < 0.05, ***p < 0.01

**Fig. 2:**
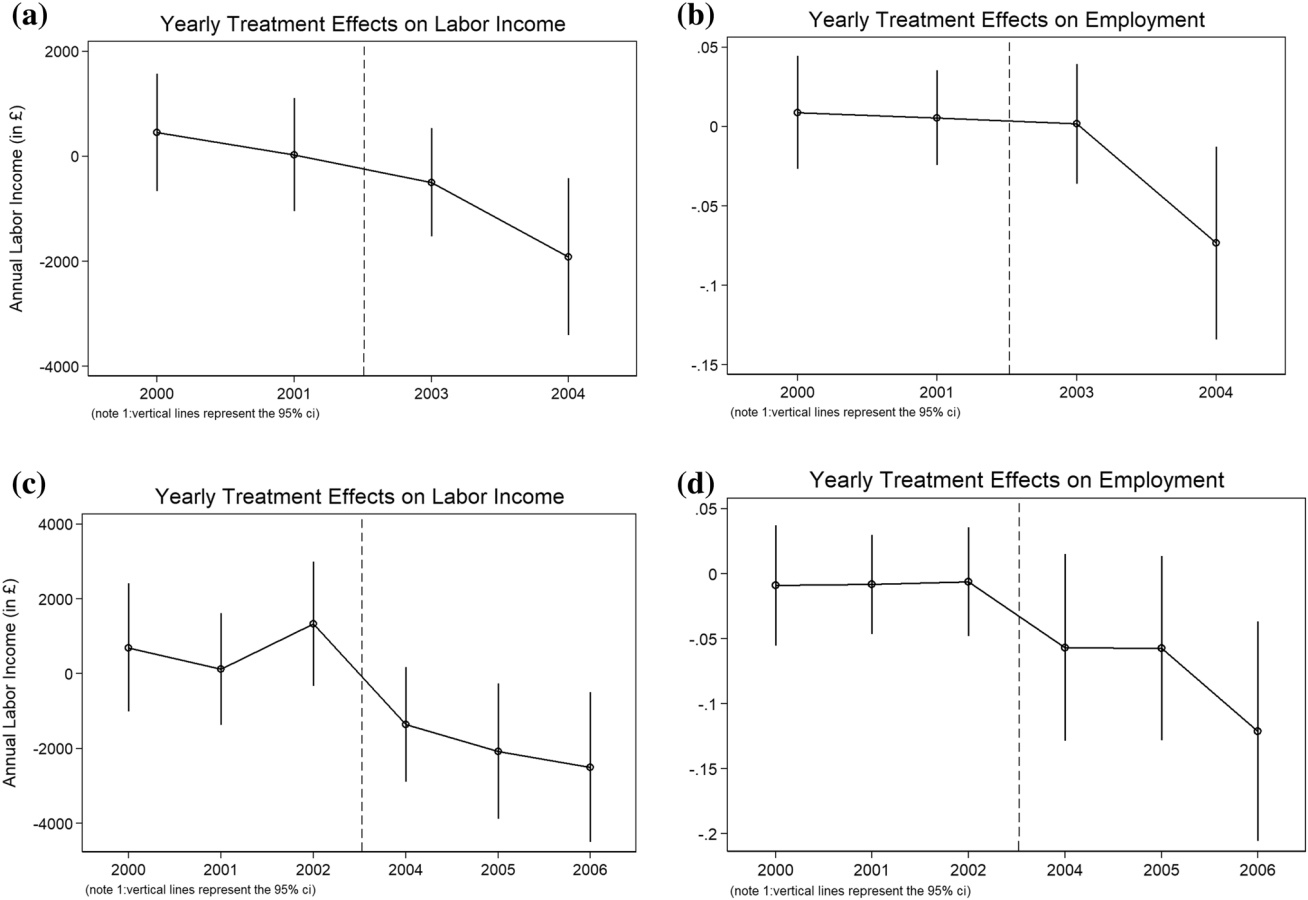
Annual treatment effects on labor income, **a** 5-year sample. **b** Annual treatment effects on employment, 5-year sample. **c** Annual treatment effects on labor income, 7-year sample. **d** Annual treatment effects on employment, 7-year sample

For all the sample periods, no statistically significant differences are estimated during the years before the onset of the health shocks. Furthermore, none of the pre-shock treatment effects that are shown in Table 5 are jointly significant. This provides suggestive evidence that the parallel trends assumption is satisfied. The estimates for both labor income employment status become larger in magnitude several years after the shocks, indicating that the effects of health shocks on labor market outcomes are persistent rather than temporary. Figure 2a–d confirm this by providing graphical representations of estimates presented in Table 5.

### Mild vs. severe health shocks

The longitudinal nature of the BHPS furthermore allows me to identify individuals who experienced large changes in self-reported health as well as others whose health status only slightly declined. Information on self-reported health status in the BHPS is provided on a scale from 1 (= excellent) to 5 (= very poor). For this analysis, I define the two types of treatments the following way for all sample lengths: (1) mild health shocks are average declines in self-reported health by at most one point on the scale between the pre- and post-shock period; (2) severe health shocks are average declines in self-reported health by more than one point on the scale between the pre- and post-shock period. Individuals in the control group are those whose average health status remained the same across both periods. Compared to the main analysis in Sect. 5.1, this specification allows using changes in the entire distribution of health status. Consistent with the main DD setup shown in Table 1, all individuals are still required to be employed throughout the pre-shock period and in the year that the shock occurred. This analysis is similar to two previous studies that test for employment effects for individuals near retirement with panel data sets. Smith [54] data from the Health and Retirement Survey (HRS) to separately test the effects of experiencing either a major or a minor health shock, while Riphahn [49] tests for the effects of labor market outcomes following a 5-point drop in health satisfaction in the German Socio-Economic Panel (GSOEP), which collects self-reported health information on a scale from 0 to 10.

Table 6 presents the results for the two levels of health shocks. As expected, the negative effects on labor income and the likelihood of being employed are substantially larger for individuals who experienced severe health shocks compared to individuals whose average health status declined by at most one point. Similar to the previous findings, the results are larger for the two longer sample periods (5 and 7 years), suggesting that the labor market effects are persistent rather than temporary. In the 7-year period, it is observable that individuals with mild health shocks significantly increase their weekly work hours, while those who experienced severe health shocks work significantly fewer hours after the health shock. Overall, the results in Table 6 point out that labor market outcomes are significantly worsened after severe health declines. However, given that labor income for those with mild shocks is reduced by £1840 in the 9-year sample (*p* < 0.01, kernel matching), the results also indicate that even relatively small health declines can negatively affect labor market outcomes of affected individuals.

**Table 6:** The effects of health shocks on labor market outcomes (average differences in health status)

	Total labor income (£ per year)		Total HH income (£ per year)		Employed		Weekly work hours	
	NN matching	Kernel matching	NN matching	Kernel matching	NN matching	Kernel matching	NN matching	Kernel matching
3-year sample								
Mild shock	− 565.58** (239.51)	− 568.12* (322.62)	− 368.59 (442.20)	− 584.43 (514.00)	− 0.0097** (0.0041)	− 0.0117** (0.0053)	− 0.03 (0.27)	0.22 (0.38)
Severe shock	− 1743.84*** (462.67)	− 1108.57* (598.55)	− 1411.01 (915.97)	− 1495.33 (1138.41)	− 0.0261** (0.0107)	− 0.0294*** (0.0111)	1.11** (0.49)	1.58** (0.75)
5-year sample								
Mild shock	− 298.32 (234.22)	− 406.03 (295.50)	372.45 (341.97)	610.45 (452.44)	− 0.0079** (0.0033)	− 0.0064 (0.0042)	0.17 (0.21)	0.12 (0.27)
Severe shock	− 1353.91*** (395.69)	− 1178.19** (515.06)	− 79.18 (768.42)	562.42 (918.08)	− 0.0373*** (0.0097)	− 0.0326*** (0.0109)	0.76 (0.51)	− 0.10 (0.66)
7-year sample								
Mild shock	− 777.23*** (254.05)	− 684.35* (379.52)	− 432.31 (399.68)	− 607.45 (578.83)	− 0.0071 (0.0044)	− 0.0012 (0.0059)	0.79*** (0.26)	1.16*** (0.36)
Severe shock	− 3697.61*** (362.84)	− 2483.74*** (547.43)	− 4366.20*** (587.85)	− 2546.32*** (1006.68)	− 0.0652*** (0.0132)	− 0.0594*** (0.0119)	− 1.52** (0.66)	− 1.90*** (0.71)
9-year sample								
Mild shock	− 1739.05*** (269.20)	− 1840.00*** (341.71)	− 3908.70*** (371.18)	− 3758.49*** (490.90)	− 0.0205 (0.0142)	− 0.0113** (0.0053)	0.25 (0.23)	0.46 (0.31)
Severe shock	− 3335.97*** (578.20)	− 3873.31*** (832.38)	− 5716.03*** (775.68)	− 7504.61*** (1361.91)	− 0.0723*** (0.0130)	− 0.0619*** (0.0140)	− 2.66*** (0.68)	− 1.74** (0.78)

Robust standard errors, cluster by individuals and based on Abadie and Imbens [1], are shown in parentheses. Income is adjusted for inflation, using the UK. Consumer price Index and 2000 as the base year

*p < 0.10, **p < 0.05, ***p < 0.01

### The effects of health conditions

For the results shown in Table 7, I use the onset of a new health condition as an alternative health shock. Given that the presence of health conditions is likely to be more objective than self-reported health status, these estimates can potentially provide additional robustness to the findings shown in Table 4 by removing concerns about the use of self-assessed health. As shown in Table 2, the number of treated individuals captured with this alternative definition of health shock is larger than for the drop in health status.

**Table 7:** Effects of health shocks on labor market outcomes (health condition)

	Total labor income (£ per year)		Total HH income (£ per year)		Employed		Weekly work hours	
	NN matching	Kernel matching	NN matching	Kernel matching	NN matching	Kernel matching	NN matching	Kernel matching
3-year sample	− 1049.24*** (364.98)	− 1068.43** (515.82)	− 2107.76*** (580.60)	− 2500.87*** (860.66)	− 0.0066 (0.0070)	− 0.0029 (0.0071)	− 0.39 (0.37)	− 0.83* (0.50)
5-year sample	− 1653.48*** (340.84)	− 1414.43*** (521.67)	− 2105.80*** (716.49)	− 3490.51*** (940.21)	0.0020 (0.0022)	0.0046 (0.0029)	− 0.83** (0.36)	− 1.15** (0.48)
7-year sample	− 3129.55*** (444.22)	− 3292.39*** (698.85)	− 3342.17*** (777.31)	− 4202.84*** (1078.69)	− 0.0025 (0.0013)	− 0.0035 (0.0025)	− 0.30 (0.39)	− 0.51 (0.55)
9-year sample	− 3482.73*** (554.06)	− 5122.64** (2017.59)	− 4097.99*** (1059.44)	− 8083.43*** (1996.55)	0.0097 (0.0074)	0.0116 (0.0152)	0.11 (0.58)	0.45 (0.94)

Robust standard errors, clustered by individuals and based on Abadie and Imbens [1], are shown in parentheses. Income is adjusted for inflation, using the UK. Consumer price Index and 2000 as the base year

*p < 0.10, **p < 0.05, ***p < 0.01

Table 7 shows that the negative effects on both labor and household income are consistent with the results from the health status shock, with all effects being statistically significant at the 1% level. The results for employment indicate the onset of a new health condition did not affect employment on the extensive margin, which again suggests that other factors explain the losses of earnings and household incomes after the health shock. The observed effects on hours worked are mixed, with three estimates finding statistically reductions in the weekly time spent working following the onset of the health condition. Overall, the results in Tables 4 and 7 provide consistent evidence that sudden health declines lead to substantial and persistent negative effects on labor earning and household income.

## Mechanisms

### The effects on health care usage

Table 8 presents estimates for the effects of health shocks on three indicators of health care usage and on the likelihood with which individuals paid for any health services out of their own pockets. The first six columns show that, as one could expect, individuals are more likely to have more than five annual doctor visits, to spend a night at the hospital and to have used any other services (e.g., physiotherapist, psychotherapist, health visitor at home) over the last 12 months. While the effects are largest in the 3-year sample, where the results capture the results in the years immediately after the health shock, the results remain relatively large and statistically significant for the longer sample periods. Given that spending a night in the hospital or frequent doctor visits means lost time at work, the observed changes in health care usage can potentially explain the earnings losses to some extent.

**Table 8:** Effects of health shocks on health care usage

	More than 5 annual doctor visits		Spent a night at hospital		Used any other health services		Paid for any health services	
	NN	Kernel	NN	Kernel	NN	Kernel	NN	Kernel
3 years	0.2329*** (0.0190)	0.2555*** (0.0204)	0.0980*** (0.0160)	0.0931*** (0.0174)	0.2403*** (0.0234)	0.2477*** (0.0282)	0.0346*** (0.0159)	0.0261*** (0.0181)
5 years	0.1742*** (0.0207)	0.1906*** (0.0204)	0.0631*** (0.0135)	0.0720*** (0.0162)	0.1397*** (0.0226)	0.1809*** (0.0283)	0.0349*** (0.0145)	0.0570*** (0.0181)
7 years	0.1794*** (0.0202)	0.2104*** (0.0218)	0.0773*** (0.0167)	0.0813*** (0.0161)	0.2058*** (0.0261)	0.2288*** (0.0308)	0.0530*** (0.0183)	0.0489** (0.0197)
9 years	0.1611*** (0.0440)	0.1774*** (0.0250)	0.0345 (0.0219)	0.0556*** (0.0174)	0.1498*** (0.0397)	0.1127*** (0.0393)	− 0.0194 (0.0161)	− 0.0175 (0.0227)

Robust standard errors, clustered by individuals and based on Abadie and Imbens [1], are shown in parentheses. Examples of health services asked for in the BHPS are usage of a physiotherapist, psychotherapist, health visitor at home and a hospital consultant. Pregnancies are excluded when examining changes in the likelihood of being a hospital in-patient

*p < 0.10, **p < 0.05, ***p < 0.01

The final two columns of Table 8 additionally provide evidence that treated individuals are more likely to pay for any health care services following the health care shock. The nearest neighbor matching results suggest that the effect is largest for the 7-year sample, again indicating that the effects on health are persistent. Given that only a small share of individuals in my samples report that they have any health care expenditures, the increase of paying for health care services of 5.30% points (*p* < 0.01) corresponds to an increase of 52.01% compared to prior to the health shock.

These observed changes in health care expenditures could furthermore explain the fact that household income reductions following health shocks are even larger than the losses in labor earnings, as previously shown in Tables 4 and 7. Other household members might reduce their work time to support the family members with health issues with their doctor visits, which goes along with increased health expenditures. While increases in health care expenditures are observable for the first three sample periods, no statistically significant effects are found for the 9-year sample.

### The effects on worker’s productivity

Another potential channel through which health shocks can affect labor market outcomes are changes in the level of work productivity. In Table 9, I show the effects on four proxies for work productivity for the sample of people who work throughout the sample period.

**Table 9:** Effects of health shocks on work productivity

	Hourly wage (£ per hour)		Work limited by health		Having difficulty to concentrate		Feeling constantly under strain	
	Nearest neighbor matching	Kernel matching	Nearest neighbor matching	Kernel matching	Nearest neighbor matching	Kernel matching	Nearest neighbor matching	Kernel matching
3 years	− 0.2933 (0.3998)	− 0.6774 (0.4684)	0.1953*** (0.0180)	0.1915*** (0.0182)	0.1806*** (0.0213)	0.1721*** (0.0178)	0.1493*** (0.0217)	0.1570*** (0.0277)
5 years	− 1.4467*** (0.1870)	− 1.3654*** (0.3897)	0.0837*** (0.0144)	0.0922*** (0.0167)	0.0809*** (0.0182)	0.0789*** (0.0239)	0.1470*** (0.0271)	0.1535*** (0.0279)
7 years	− 0.8068** (0.3794)	− 0.0947 (0.3334)	0.1060*** (0.0158)	0.1007*** (0.0200)	0.1833*** (0.0240)	0.1643*** (0.0241)	0.1956*** (0.0239)	0.1677*** (0.0291)
9 years	− 2.0683*** (0.2474)	− 2.0709*** (0.7561)	0.0860*** (0.0277)	0.0857*** (0.0234)	0.0230* (0.0120)	0.0362** (0.0180)	0.1032*** (0.0338)	0.0712*** (0.0338)

Robust standard errors, clustered by individuals and based on Abadie and Imbens [1], are shown in parentheses

*p < 0.10, **p < 0.05, ***p < 0.01

First, I examine whether health shocks affect the hourly wages of individuals who remain in the workforce. The DD results provide evidence that wage rates declined substantially for workers who experienced adverse health events compared to those who did not. While the estimates for the 3-year period are relatively small and imprecisely estimated, I find that hourly wages are reduced by £2.07 (*p* < 0.01) when analyzing the 9-year sample. These estimates suggest that individuals who remain in the workforce experience less wage growth than those in the control group following a health shock. One potential explanation for this could be that they are either not able to perform the same tasks or take longer to complete the same tasks as compared to prior to the onset of the health shock. The remaining columns of Table 9 examines several proxies for labor productivity that can provide more evidence on how work performance can be affected by health shocks.

The next two columns show that treated workers are significantly more likely to report that their health is limiting their work. Similar to changes in health care usage, the effects are largest in the year after the health shock. Using the 3-year sample, I observe a 19.53% point increase in the likelihood of reporting health-related work limitations (*p* < 0.01). While the effects are smaller for the three longer sample periods, they still show statistically significant increases (*p* < 0.01). The other two proxies of work productivity I examine are reporting having difficulties to concentrate [5] and being constantly under strain [27]. The DD matching estimates obtained for these two outcomes provide additional evidence that reductions in work productivity might explain the losses of labor income to some extent. Again, the effects are quite large and remain persistent across the different sample periods. Overall, the results in Table 9 suggests that individuals who suffered from a sudden health shock are less likely to perform the same tasks compared to prior to the health shock.^6^

## Heterogeneous effects

In a number of additional specifications, I examine whether the effects of sudden health declines on labor earnings differ across subgroups of the population. Table 10 presents nearest neighbor DD matching results across gender, education level, job classifications, and age. Using data from the Netherlands, García-Gómez et al. [24] find that health shocks have larger effects on the income of men, which they relate to the fact that males are accounting for greater shares of household earnings. Using longitudinal data from the USA, Charles [11] furthermore provides evidence that the effects of health shocks on earnings are increasing with age. He provides two explanations for this: (1) older persons have accumulated more human capital that can be destroyed by negative health events; (2) any subsequent recovery in earnings will be weaker for older individuals.

**Table 10:** heterogeneous effects of health shocks on earnings (health status)

	Total labor income (£ per year)			
	3-year	5-year	7-year	9-year
Panel A: gender				
Male	− 2535.31*** (615.30)	− 6576.00*** (608.28)	− 5552.71*** (721.11)	− 3248.65*** (517.66)
Female	− 615.02 (584.40)	− 1351.57*** (506.31)	− 1310.93*** (446.87)	− 2131.27*** (649.38)
Panel B: education				
Advanced degree	− 2157.03*** (523.83)	− 3166.42*** (572.38)	− 3255.57*** (555.75)	− 3151.08*** (863.72)
Basic degree/low education	− 935.79** (418.38)	− 1253.72*** (367.72)	− 2592.76*** (497.77)	− 2771.53*** (404.12)
Panel C: job classification				
Managerial/professional job	− 1966.34*** (722.16)	− 3411.18*** (714.28)	− 3250.00*** (965.94)	− 6507.92*** (698.68)
Skilled labor	150.67 (386.22)	− 2066.42*** (447.94)	− 433.09 (554.37)	− 2436.33*** (680.92)
Unskilled labor	289.68 (844.24)	− 1349.32*** (459.43)	− 91.13 (1142.81)	− 2079.77*** (475.26)
Panel D: age				
Below 40 years	− 1928.86*** (419.86)	− 3845.26*** (599.12)	− 2435.64*** (545.65)	− 3582.65*** (878.89)
At least 40	− 1110.15* (650.25)	− 3836.11*** (566.79)	− 2944.72*** (885.39)	− 2415.16*** (839.80)

Robust standard errors, clustered by individuals and based on Abadie and Imbens [1], are shown in parentheses. Income is adjusted for inflation, using the UK. Consumer price Index and 2000 as the base year

*p < 0.10, **p < 0.05, ***p < 0.01

My findings in Panel A confirm the results by García-Gómez et al. [24]. For all four sample periods, the effects of health status declines on earnings are substantially larger for male individuals. In the 5-year sample period, a health shock is shown to reduce labor earnings of men by £6576.00, compared to a reduction of earnings of £1351.57 for women (both *p* < 0.01). Similar to García-Gómez et al. [24], I find that men have substantially higher pre-shock earnings than women, which could explain the different effects to some extent. Panels B and C additionally provide evidence that health shocks have stronger effects on labor market outcomes of individuals with higher education levels and for those who work in managerial or professional jobs. Again, differences, in income prior to the health shock can potentially explain the larger effects for these two groups. Finally, the results in Panel D do not indicate that the effects differ largely across age groups.

## Discussion and conclusions

The findings in this study provide evidence that health shocks significantly affect the labor market outcomes of individuals in the UK for several years after the decline in health. García-Gómez et al. [24] suggest that negative effects of health shocks on labor markets can exist either due to incentives created by disability benefits or due to labor market institutions constraining the responsiveness of wages to reduced productivity. Given that the disability benefit scheme in the UK provides benefits at a flat rate, it creates very little incentives for individuals to voluntary reduce their employment compared to other countries, which provide disability benefits that are closely tied to previous earnings [58]. This suggests that the observed reductions in labor market participation following health shocks are not driven by incentives provides by disability benefits.

This paper shows that the declines are not entirely driven by changes in employment status, but are also observable for individuals who remained employed. Additionally, the study provides first evidence that changes in work productivity is a mechanism through which health shocks lead to lower labor earnings. Individuals who suffer sudden health declines are shown to be limited in work-related activities and to have difficulties concentrating in the following years, suggesting lower levels of work productivity and the inability to complete the same tasks the were able to perform before the health shock. Given that my results suggest that the negative effects on work productivity are still observable several years after the health shock, policymakers and employers should think about ways how the reintegration of employees can be improved and significant productivity loss can be avoided.

Additionally, despite the provision of universal health care through the NHS in the UK, I find significant increases in the likelihood with which individuals pay for health care services following the onset of a health shock. A likely explanation for this might be that individuals want to forego long waiting times before receiving treatment and decide to pay for private care. The NHS has been dealing with the issue of long waiting times for several decades. Using official data from the NHS, Murray [42] shows that 2015 marked the first year since the introduction of the NHS in which the standard that at least 92% of patients receive their treatment within 18 weeks was not achieved, suggesting that the issue is becoming worse over time. Thus, policymakers should discuss ways on how to stop the trend of increasing waiting times since they can cause further harm to individuals suffering health shock by affecting household incomes, financial stress and overall well-being in the following years.

Previous data on the overall level of health for the UK population is mixed. A report of the Economist Intelligence Unit that compares healthcare inputs and outcomes across 166 countries ranked the UK 23rd in terms of performance [19]. A report by the Commonwealth Fund in 2014 places the UK second to last in the ‘healthy lives’ category, which uses indicators of population health outcomes, including mortality, infant mortality and life expectancy [17]. Evidence from the Global Burden of Disease Study shows that the leading risk factors for premature death in the UK are linked to lifestyle, in particular to dietary risk, tobacco smoking, high blood pressure, and alcohol consumption [30]. Given that these causes are preventable, campaigns to promote healthier lifestyles and make people more aware of health risks could be successful in improving health outcomes and in preventing the onset of sudden health shocks for people in the workforce. Since 1998, the UK government has had success in reducing smoking of the population from 28 to 18% in 2015. It has done so by changing taxation, by increasing public awareness of the harm caused by smoking and by helping people quit [6]. These policy interventions should be continued and expanded to other lifestyle choices that cause harm to people’s health.

## Electronic supplementary material

Below is the link to the electronic supplementary material.

Supplementary material 1 (DOCX 197 KB)

## Footnotes

1 Taylor (1998) provides a full description of the sampling strategy applied in the initial wave in order to design a nationally representative sample of the British population.

2 The analysis is conducted using the “teffects psmatch” command in Stata, which incorporates the findings by Abadie and Imbens [3] and takes into account potential estimation errors in the propensity score due to the fact that the propensity scores were estimated in the first stage. In an earlier paper, Abadie and Imbens [2] show that bootstrapping, a technique that had previously often been used to obtain standard errors for propensity score matching estimators, is generally not valid for matching estimators.

3 Table 3 shows balancing test results for the Nearest Neighbor matching analysis. The results are consistent for the kernel matching analysis. These results are not shown, but are available upon request.

4 Income results are adjusted for inflation using the U.K. Consumer Price Index and 2000 as the base year.

5 Appendix Table A3 furthermore shows DD matching estimates when lagged health status is included as a covariate to obtain the propensity score values. The results are consistent with the main results from Table 3, providing further evidence that negative health events affect labor market outcomes.

6 All results in Tables 8 and 9 are obtained using the drop in health status as the health shock. Similar to the previous section, the results remain consistent when using the onset of a health condition as the health shock. These additional results are not shown in the paper, but are available upon request.
