# Supplementary material for: The effects of health shocks on labor market outcomes: evidence from UK panel data
Source: Eur J Health Econ. 2018 Jun 6;20(1):83–98. doi: 10.1007/s10198-018-0985-z (PMC6394599; doi:10.1007/s10198-018-0985-z)

| Table A1: Descriptive Statistics - Attrition Sample | | | | | | |
| --- | --- | --- | --- | --- | --- | --- |
|  | **5-Year Sample** | | **7-Year Sample** | | **9-Year Sample** | |
|  | Dropped out of sample | Remained in sample | Dropped out of sample | Remained in sample | Dropped out of sample | Remained in sample |
| *Health Status* |  |  |  |  |  |  |
| Excellent | 0.3684 | 0.3563 | 0.3856 | 0.3832 | 0.4142 | 0.3975 |
|  | (0.4824) | (0.4789) | (0.4868) | (0.4858) | (0.4926) | (0.4894) |
| Good | 0.5921 | 0.5681 | 0.5702 | 0.5574 | 0.5495 | 0.5494 |
|  | (0.4915) | (0.4953) | (0.4952) | (0.4965) | (0.4976) | (0.4976) |
| Fair | 0.0333 | 0.0617 | 0.0350 | 0.0484 | 0.0288 | 0.0435 |
|  | (0.1795) | (0.2406) | (0.1834) | (0.2142) | (0.1672) | (0.2039) |
| Poor | 0.0050 | 0.0122 | 0.0076 | 0.0092 | 0.0056 | 0.0082 |
|  | (0.0705) | (0.1096) | (0.0867) | (0.0945) | (0.0746) | (0.0902) |
| Very Poor | 0.0012 | 0.0017 | 0.0017 | 0.0018) | 0.0020 | 0.0014 |
|  | (0.0345) | (0.0413) | (0.0407) | (0.0447) | (0.0447) | (0.0375) |
|  |  |  |  |  |  |  |
| *Employed* | 0.7780 | 0.8258 | 0.8273 | 0.8425 | 0.8369 | 0.8643 |
|  | (0.4157) | (0.3793) | (0.3780) | (0.3643) | (0.3695) | (0.3425) |
|  |  |  |  |  |  |  |
| *Total Annual Labor Income (in £)* | 16,135.07 | 18,483.95 | 18,353.51 | 19,918.16 | 20,055.19 | 21,055.19 |
|  | (14,619.31) | (16,782.78) | (13,255.28) | (17,237.20) | (18,342.15) | (18,342.15) |
|  |  |  |  |  |  |  |
| *Total Annual Household Income (in £)* | 33,030.26 | 37,649.83 | 37,364.41 | 40,192.18 | 39,884.20 | 42,172.82 |
|  | (22.371.34) | (24.942.26) | (21,988.04) | (25,817.28) | (24,516.43) | (27,586.53) |
|  |  |  |  |  |  |  |
| *% dropped out of sample* | 14.58% | | 13.93% | | 15.30% | |
|  |  |  |  |  |  |  |

| Table A2: Effects of Health Shocks on Log of Income | | | | |
| --- | --- | --- | --- | --- |
|  | **Log (Total Labor Income)** | | **Log (Total HH Income)** | |
|  | NN matching | Kernel matching | NN matching | Kernel matching |
|  |  |  |  |  |
| 3 Year Sample | -0.0599** | -0.0676** | -0.0689*** | -0.0644** |
|  | (0.0250) | (0.0341) | (0.0200) | (0.0300) |
|  |  |  |  |  |
| 5-Year Sample | -0.0890*** | -0.1408*** | -0.0860*** | -0.1025*** |
|  | (0.0339) | (0.0425) | (0.0305) | (0.0197) |
|  |  |  |  |  |
| 7-Year Sample | -0.0407 | -0.0707* | -0.0552*** | -0.0500* |
|  | (0.0293) | (0.0388) | (0.0185) | (0.0296) |
|  |  |  |  |  |
| 9-Year Sample | -0.1776* | -0.0522 | -0.1041*** | -0.1435*** |
|  | (0.1019) | (0.0588) | (0.0297) | (0.0430) |
|  |  |  |  |  |

*Notes*: Robust standard errors, clustered by individuals and based on Abadie and Imbens (2006), are shown in parentheses. Income is adjusted for inflation, using the U.K. Consumer price Index and 2000 as the base year. ^*^ *p* < 0.10, ^**^ *p* < 0.05, ^***^ *p* < 0.01.

| Table A3: Effects of Health Shocks on Labor Market Outcomes  (controlling for lagged health status) | | | | |
| --- | --- | --- | --- | --- |
|  | **Total Labor Income (£ per year)** | | **Employed** | |
|  | NN matching | Kernel matching | NN matching | Kernel matching |
|  |  |  |  |  |
| 5-Year Sample | -1,741.57*** | -1,889.37*** | -0.0297* | -0.0171 |
|  | (610.89) | (639.60) | (0.0155) | (0.0126) |
|  |  |  |  |  |
| 7-Year Sample | -1,817.65*** | 70.79 | -0.0579 | -0.0357*** |
|  | (574.80) | (801.74) | (0.0449) | (0.0136) |
|  |  |  |  |  |
| 9-Year Sample | -5,186.15*** | -5,120.58*** | -0.0157 | 0.0032 |
|  | (793.72) | (986.17) | (0.0213) | (0.0192) |
|  |  |  |  |  |

*Notes*: Robust standard errors, clustered by individuals and based on Abadie and Imbens (2006), are shown in parentheses. Income is adjusted for inflation, using the U.K. Consumer price Index and 2000 as the base year. ^*^ *p* < 0.10, ^**^ *p* < 0.05, ^***^ *p* < 0.01.

Figure A1(a): Density of Propensity Scores with Controls for Lagged Health, 5-Year Sample


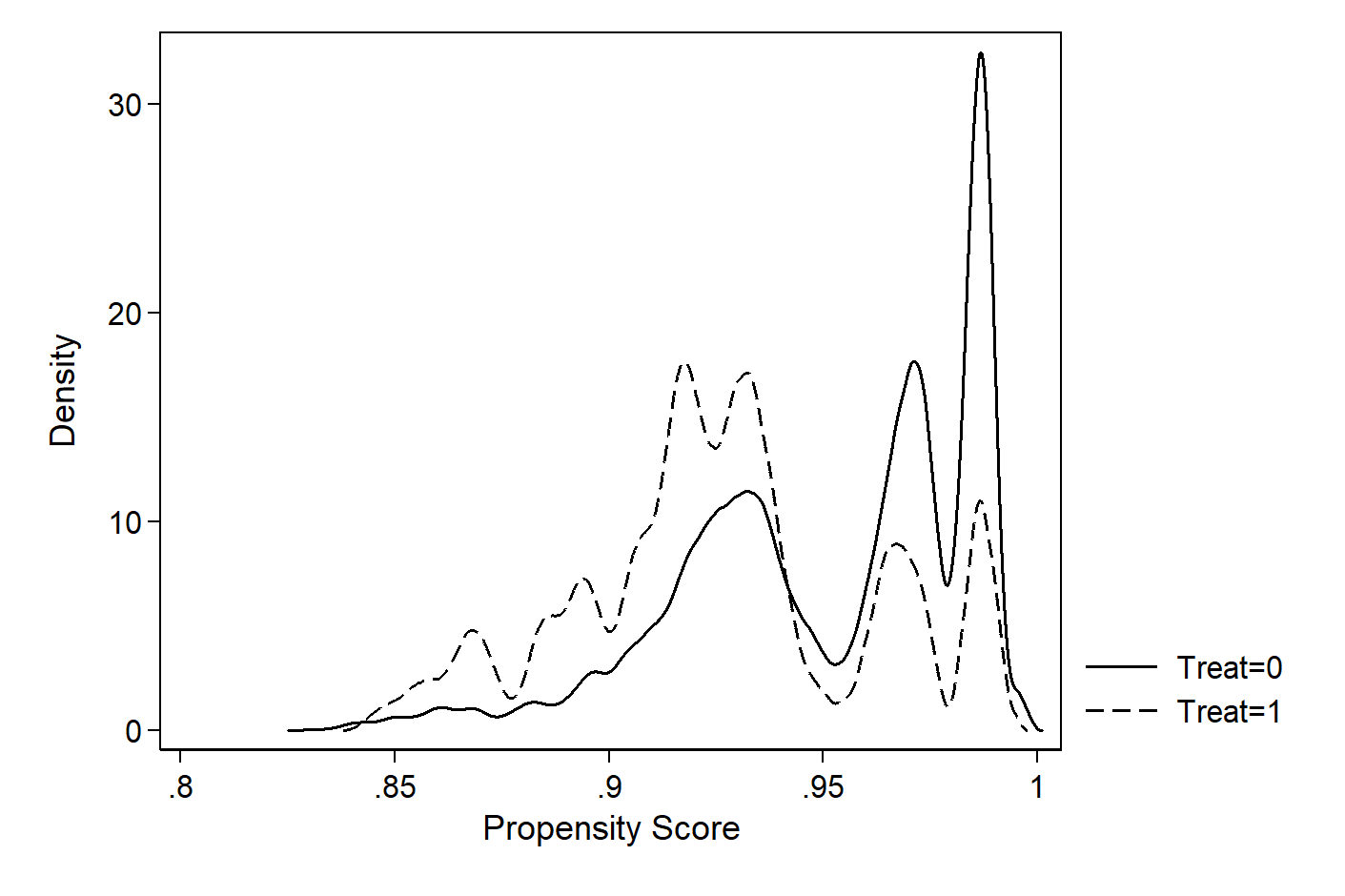


Figure A1b): Density of Propensity Scores with Controls for Lagged Health, 7-Year Sample


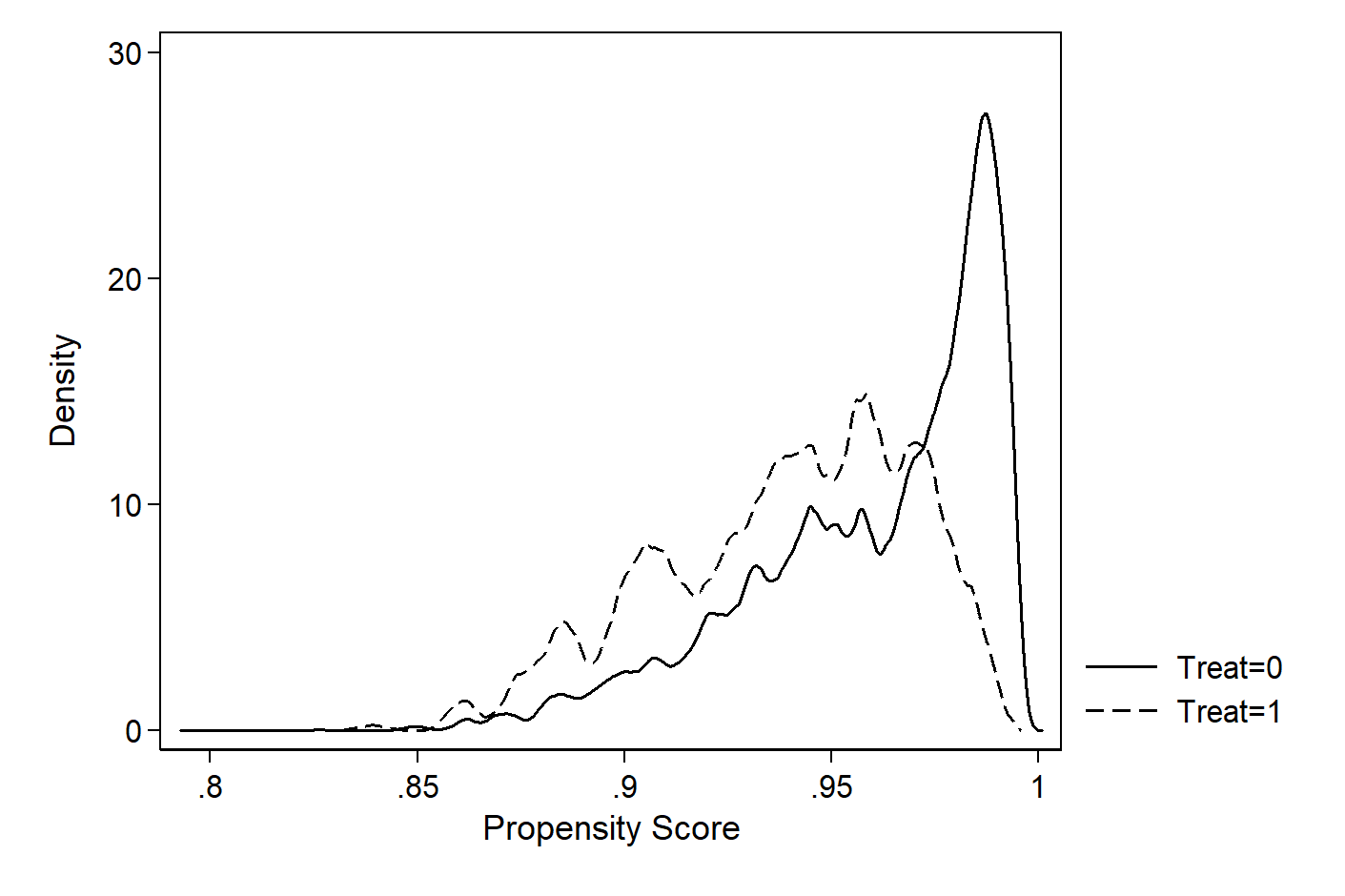


Figure A1(c): Density of Propensity Scores with Controls for Lagged Health, 9-Year Sample


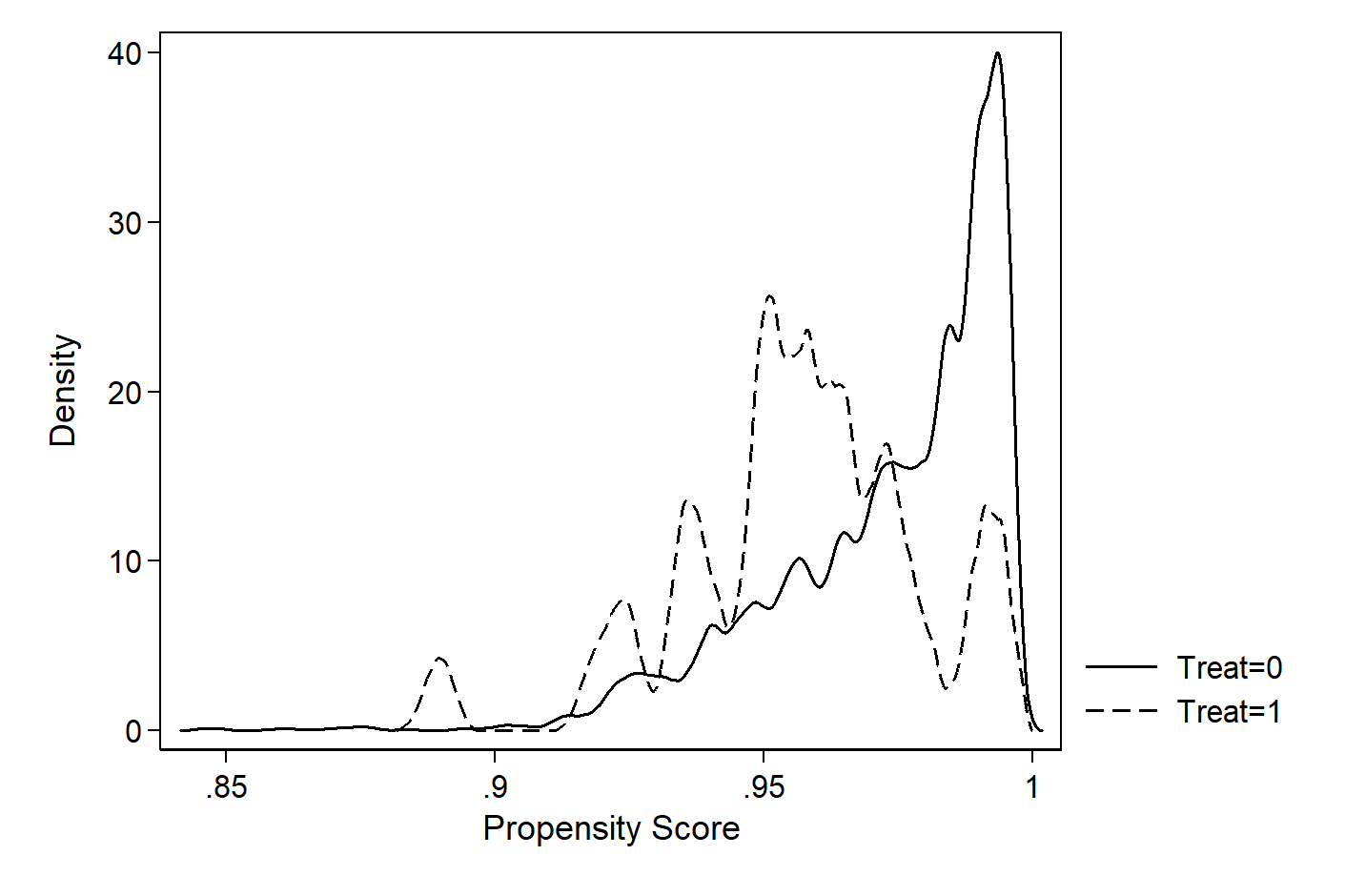

Supplement: Supplementary file 1 — Supplementary material 1 (DOCX 197 KB) [file 10198_2018_985_MOESM1_ESM.docx]
